# Traumatic experiences and mental health consequences among child survivors of the 2008 Sichuan earthquake: a community-based follow-up study

**DOI:** 10.1186/1471-2458-13-104

**Published:** 2013-02-05

**Authors:** Zhaobao Jia, Lizheng Shi, Guangfeng Duan, Weizhi Liu, Xiao Pan, Yingyao Chen, Wenhua Tian

**Affiliations:** 1 Department of Health Service Management, Second Military Medical University, Shanghai, 200433, China; 2 Department of Health Systems Management, School of Public Health and Tropical Medicine, Tulane University, New Orleans, USA; 3 Department of Psychology, Second Military Medical University, Shanghai, China; 4 Department of Hospital Management, Fudan University, Shanghai, China

**Keywords:** Posttraumatic stress disorder, Depression, Traumatic experiences, Child, Earthquakes

## Abstract

**Background:**

The study was implemented to examine the relationship between traumatic experiences and longitudinal development of mental health for children and adolescents who survived the 2008 Sichuan earthquake.

**Methods:**

Using the method of multistage systematic sampling, 596 children aged between 8 and 16 years were randomly selected from severely affected areas of the earthquake. These children were interviewed with standardized instruments of posttraumatic stress disorder (PTSD) and depression at the 15th month after the earthquake, and re-interviewed at the 36th month.

**Results:**

From the initial to the follow-up assessments, there were no significant changes in both PTSD and depression scores. In addition, no significant change was found on the overall prevalence rates of the symptoms: from 12.4% to 10.7% for PTSD, from 13.9% to 13.5% for depression, and from 4.2% to 4.7% for their co-occurrence. The study also indicated that the earthquake might have a delayed impact on the psychosocial functioning of children and adolescents who were not directly affected by the disaster.

**Conclusions:**

For child and adolescent survivors of the earthquake, symptoms of PTSD and depression seemed to persist over time. The finding that children reduced their use of mental health services raised great concerns over how to fulfill the unmet psychological needs of these children. More mental health interventions should be allocated to children who had elevated risk for developing persistent course of the symptoms.

## Background

Over the past decade, Asia has been the continent frequently and severely affected by earthquakes [1]. From the 225,000 deaths caused by the 2004 Indian Ocean earthquake and tsunami [2], to 73,276 deaths by the 2005 Pakistan earthquake [3], 69,200 deaths by the 2008 China Sichuan earthquake [4], and 15,839 deaths by the 2011 Great East Japan earthquake [5], Asian countries suffered not only giant losses from the instantaneous and devastating blow of the disaster, but critical consequences from the longstanding post-earthquake adversities, e.g., the dysfunction of public health systems. As an essential component of public health, continuing attention should be paid to the mental health of the survivors because psychological impact may last for many years after the event [6-9]. However, little research has prospectively examined the psychological sequelae in Asian socioeconomic context, especially for children and adolescents.

There are theoretical and practical reasons to explore this issue. On one hand, empirical literature on the association between children’s disaster-related traumatic experiences and mental health mostly drew samples from schools [8,10-14]. This sampling method, however, is not suitable for research conducted in low-income settings of Asia, where there is a reported substantial proportion of school-aged children dropouts [15,16]. In addition, most of the longitudinal studies conducted in Asia were accomplished in a short time period, often in a year or several months [17,18], which might result in the underrepresentation of psychological development in the subjects. In the study we adopted a community-based design, an approach that is increasingly used to examine the environmental and social determinants of health [19], so that we could assess the psychological development of child survivors in a two-year period. Certainly, this research would enrich the literature of post-disaster studies in Asia. On the other hand, as psychological support is an indispensible investment to prevent mental health crisis after natural disasters, it would be beneficial to screen out children with certain characteristics that are associated with vulnerability to psychological symptoms, so that appropriate and cost-effective interventions would be allocated to them.

Therefore, by a community-based follow-up design, this study aimed to investigate the relationships between various traumatic experiences and the longitudinal development of Posttraumatic Stress Disorder (PTSD) and depression among child and adolescent survivors of the 2008 Sichuan earthquake.

## Methods

### Subjects and design

Two assessments were carried out among child and adolescent survivors, 15 and 36 months after the earthquake. A household was used as the primary sampling unit and was defined as any group of persons who lived together and shared food and bedding, etc. Before sampling, a power analysis to determine an appropriate sample size was based on symptoms of PTSD. Since the literature showed that the post-disaster prevalence rates of PTSD varied between 10% and 22% among East-Asia children [11,14,18], 10% was used to calculate the sample size as a conservative approach. Assuming a design effect of 1.5, we estimated that 576 participants were required to obtain 3.0% precision around the prior-specified 10% prevalence rate of PTSD. The final anticipated sample size was adjusted to 1,145 households, after considering the proportion of households with a child from 8 to 16 years of age and a potential 20% non-response rate.

**Figure 1:**
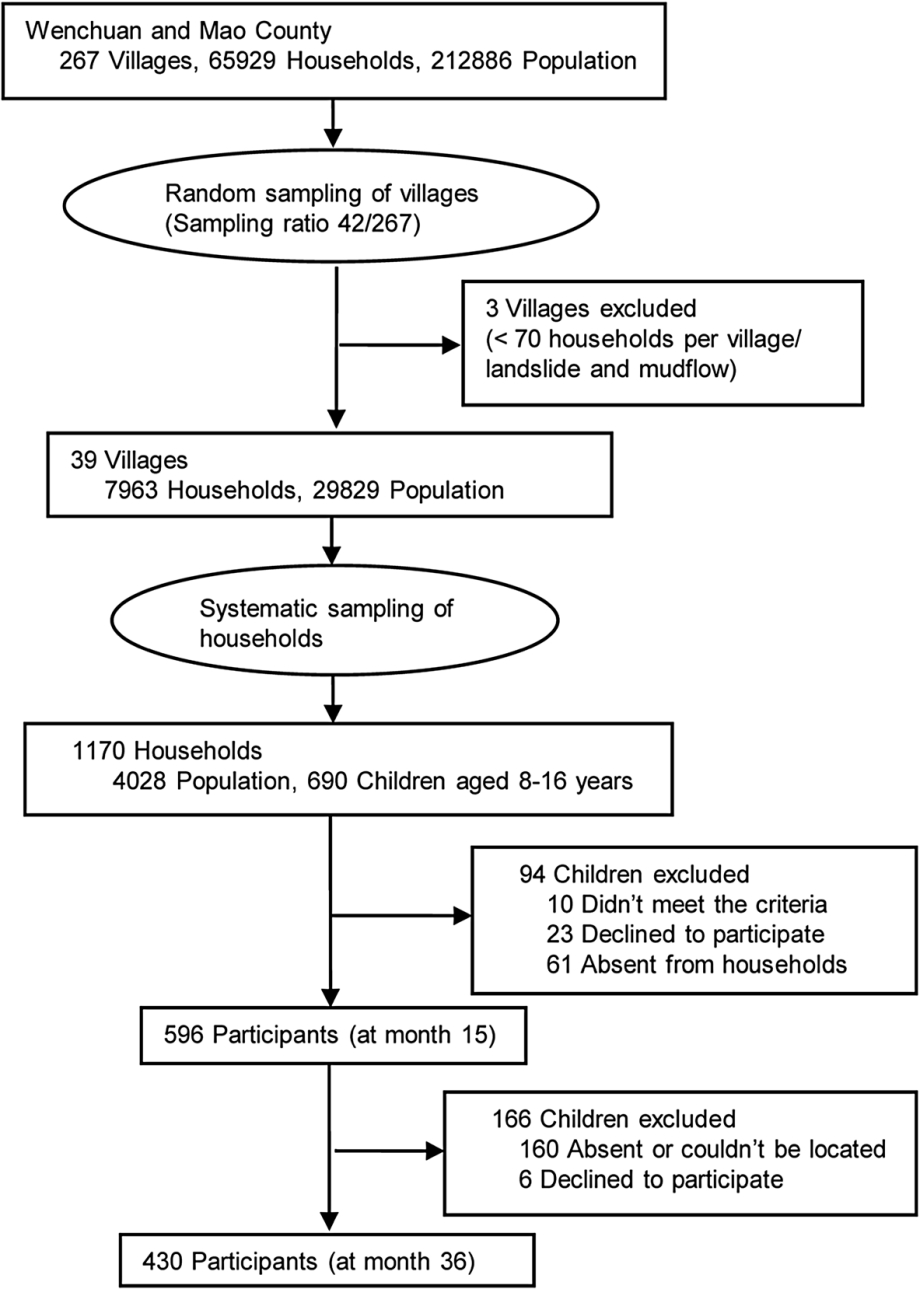
Sampling strategies for child and adolescent survivors of 2008 sichuan earthquake, China.

The study employed a multi-stage systematic sampling design to select participants in the severely affected areas of the earthquake. Details of the sampling procedure were depicted in Figure 1. In brief, two of the ten severely earthquake-affected sub-districts declared by the government, including 267 villages and over 65,900 households, were selected as the frame. After excluding villages with a very small number of households and villages with possible geological dangers, such as landslide, 39 villages were used as potential sampling sites. Within each available village, systematic sampling was performed. Households were numbered according to registration information and were systematically selected. But for a number of villages where many people no longer resided at their homes, systematic sampling was not feasible; non-random sampling methods were supplemented. Finally, a total of 1,170 households were recruited, which included 690 families with at least one child aged between 8 and 16 years.

### Data collection

Our psychologists and eight social science graduate students performed the interview. Before the assessments, interviewers were called together to review the questionnaire and achieve agreement on the explanation of each item. Exclusion criteria were absence from the affected area during the earthquake and severe learning deficiency. With the help from key informants (e.g., parents, adult neighbors, school teachers, etc.), children and adolescents with pre- or post-earthquake traumatic experiences were also excluded. When more than one child was eligible for study in a household, one child was selected according to the Kish method [20]. With the help of village, township and county-level school staff, we identified the children who were present at school at the time of the home visit. Due to the high illiteracy rate in the surveyed area, only verbal consents were sought from their guardians and then carefully recorded by the interviewers in field files after standard and complete descriptions of the study. Then each participant completed the questionnaire independently, and was presented a small gift upon its completion. A week later, almost ten percent of participants who were originally involved in the questionnaire interview were reassessed by our psychologists based on DSM-IV criteria.

### Instruments

Standardized instruments were used to measure the severity of PTSD and depression in the subjects. Symptoms of PTSD were evaluated using the self-report Child PTSD Reaction Index [21,22]. The instrument has been frequently used to assess traumatized children after major disasters and catastrophic violence, and has satisfactory psychometric properties in a Chinese context [4]. It contains 20 items rated on a 5-point Likert scale ranging from 0 (“none”) to 4 (“most of the time”), making the total score of the Child PTSD Reaction Index ranging from 0 to 80. Comparisons of Child PTSD Reaction Index scores with clinical diagnosis of PTSD have suggested the following guidelines: (1) 0–11 = suspect, 12–24 = mild, 25–39 = moderate, 40–59 = severe, 60–80 = very severe; (2) high level of agreement between a score > = 40 and diagnosis of PTSD based on DSM-IV criteria [22].

The Children’s Depression Inventory (CDI), which has been widely used to measure depression in children and adolescents in epidemiological studies [23,24], was employed to assess symptoms of depression among the participants. It contains 27 items with a self-rating scale ranging from 0 to 2 that yields a total score ranging from 0 to 54. Children with a score of 20 or above were classified as having symptoms of depression [24]. The instrument has been validated in Chinese children with Cronbach’s α ranging from 0.85 to 0.89, and test-retest reliability ranging from 0.75 to 0.85 [23,25-27].

Perceived social support was measured by the adapted Multidimensional Scale of Perceived Social Support (MSPSS) [28,29]. It contains 12 questions with a self-rating scale ranging from 0 to 4 that yields a total score ranging from 0 to 48. A Total MSPSS Score was obtained by summing across all items, with a higher score indicating more perceived social support. Hong and his colleagues have demonstrated its feasibility in China [30].

Adapted from an existing disaster exposure scale [10], the earthquake-related event exposure measure was the sum of 11 events that could occur during or after the earthquake. All these questions were coded into yes/no items, and were categorized into three groups: objective experience, earthquake-related loss, and subjective experience. Positive responses to these items were summed for analyses in each category. For mental health service utilization measures, we adopted the methodology of National Comorbidity Survey [31]. We asked participants whether they received counseling from a helping professional (e.g., psychologist, psychiatrist, physician, trained teacher, etc.) for emotional problems and whether the counseling was related to the earthquake.

To minimize potential bias, the questionnaire was verified for accuracy and comprehensibility by local mental health professionals. Questions that might be misunderstood were identified, and the best equivalent local expressions were used. The Ethics Committee of the Second Military Medical University approved the consent procedures and instruments used in the study.

### Data analysis

Means, standard deviations, frequencies and percentages were calculated for descriptive data analysis. *T*-tests were used to evaluate differences in continuous variables, and Chi-square tests were used to test for significance in categorical characteristics. Point estimates and 95% confidence intervals (CI) for prevalence rates of PTSD and depression were estimated overall as well as stratified by demographics and earthquake-related experiences. Kappa coefficients were used to test the agreement on the instrument-based and DSM-IV-based clinical diagnosis of PTSD and depression. To examine the relationships between perceived social support and symptoms of PTSD and depression, Pearson correlations were computed. Linear regression analyses were performed to examine the relative contribution of each independent variable to the severity of PTSD and depression. These variables were selected either because they were associated with the symptoms, or because previous research and *a priori* theoretical judgment have indicated that they should be included in the models. Two-tailed p < 0.05 was considered statistically significant. Data were analyzed using SPSS version 17.0 (SPSS Inc, Chicago, Ill).

## Results

A total of 596 participants were included in the initial assessment, with a response rate of 86.4% (596/690). In the follow-up assessment, 160 children were absent or couldn’t be located, and another 6 children refused to participate, hence only 430 children were retained in the follow-up assessment, which accounted for 72.1% (430/596) of the original sample. The sample at baseline consisted of 297 boys (49.8%) and 299 girls (50.2%) between the ages of 8 and 16 years, with mean (SD) age at 11.5 (2.1) and 11.4 (2.2) years, respectively. Compared with children from Wenchuan, those from Mao were more likely to report that they were in serious danger, that their houses had been seriously damaged, that they felt extremely anxious about their own lives, and that they felt scared that family members or significant others would die or be seriously injured (Table 1).

**Table 1:** Participants’ characteristics at the 15th Month, stratified by residence

	**No (%). of participants**			
**Characteristics**	**Total**	**Wenchuan**	**Mao**	**P**
Demographics				
Age, y				
8-12	307 (51.5)	134 (54.7)	173 (49.3)	0.19
13-16	289 (48.5)	111(45.3)	178 (50.7)	
Sex				
Boys	297 (49.8)	114 (46.5)	183 (52.1)	0.18
Girls	299 (50.2)	131 (53.5)	168 (47.9)	
Ethnicity				
Han	227 (38.1)	146 (59.6)	81 (23.1)	< 0.001
Ethnic minorities^a^	369 (61.9)	99 (40.4)	270 (76.9)	
Earthquake-related experiences				
Having been in serious danger (Eq1)	297 (49.8)	104 (42.4)	193 (55.0)	0.003
Having been seriously injured (Eq2)	58 (9.7)	29 (11.8)	29 (8.3)	0.15
Having family members or friends seriously injured (Eq3)	101 (16.9)	41 (16.7)	60 (17.1)	0.91
Having witnessed someone being killed or seriously injured (Eq4)	244 (40.9)	108 (44.1)	136 (38.7)	0.19
Having lost family members (Eq5)	68 (11.4)	32 (13.1)	36 (10.3)	0.29
Having lost significant others (Eq6)	145 (24.3)	58 (23.7)	87 (24.8)	0.76
Having one’s house seriously damaged (Eq7)	425 (71.3)	150 (61.2)	275 (78.3)	< 0.001
Having lost important belongings (Eq8)^b^	244 (40.9)	97 (39.6)	147 (41.9)	0.58
Having felt extremely anxious about one’s own life (Eq9)	439 (73.7)	157 (64.1)	282 (80.3)	< 0.001
Having felt scared that family members or significant others would die or be seriously injured (Eq10)	473 (79.4)	170 (69.4)	303 (86.3)	< 0.001
Having felt guilt concerning someone’s death or injury (Eq11)	220 (36.9)	80 (32.7)	140 (39.9)	.07

^a^ Ethnic minorities refer to non-Han population in China, which account for a larger population in the survey areas than Han people. ^b^ Important belongings refer to physical possessions of sentimental value to the child.

**Figure 2:**
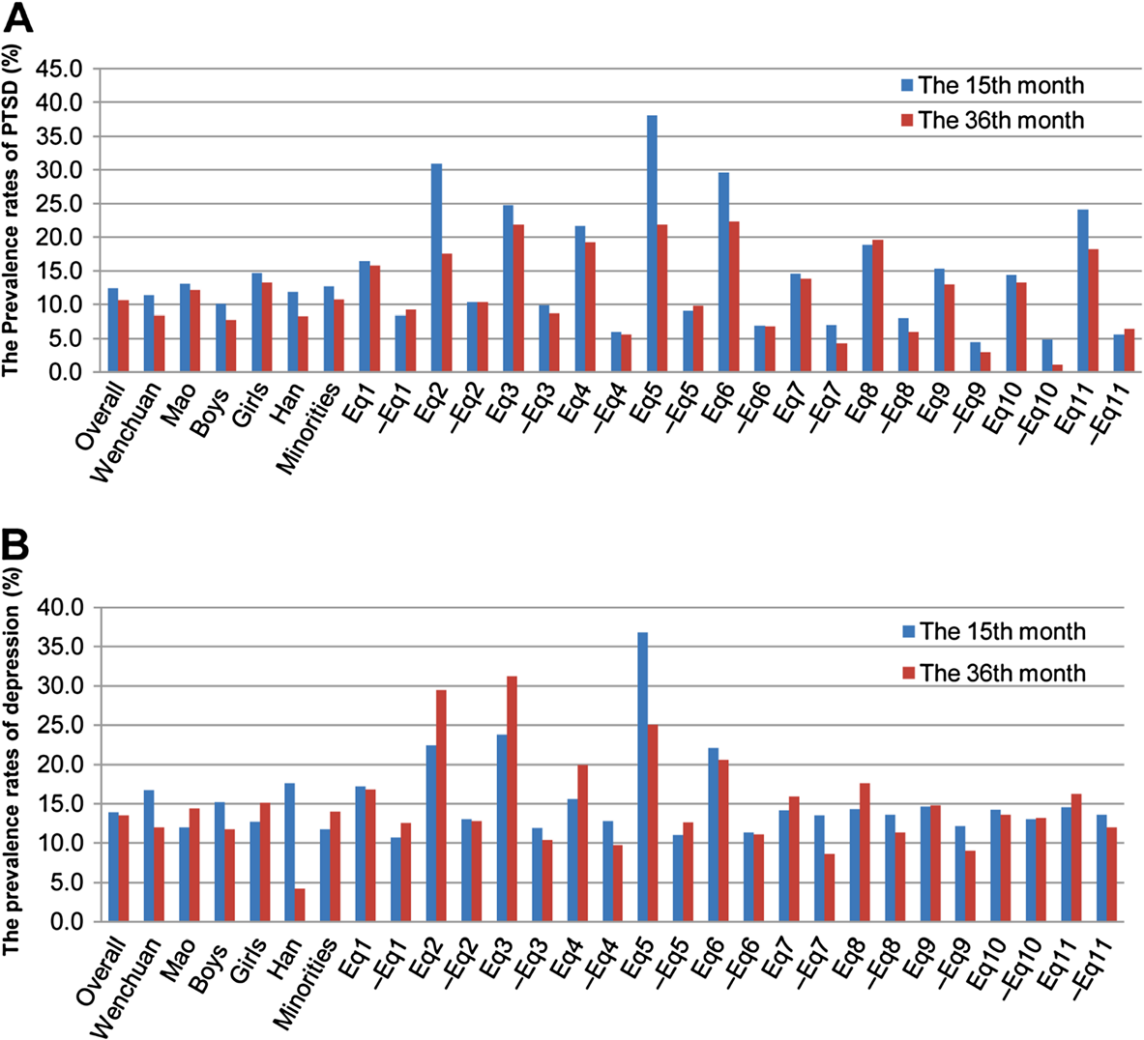
**The prevalence rates of PTSD and depression among participants with different demographics and traumatic experiences**^**a**^.^ a^ Eq1–Eq11 refer to earthquake-related experiences (see Table 1). The mark “–” before the Eq series represents children without the specific experience.

Empirically derived cutoff scores for both the Child PTSD Reaction Index and CDI were used to estimate the prevalence rates of PTSD and depression. High agreement was found between the instrument-based and DSM-IV-based clinical diagnosis of PTSD and depression, with Kappa coefficients at 89.8% and 91.2% in the initial assessment, and 87.5% and 89.5% in the follow-up assessment, respectively. In the study, the overall prevalence rates of the symptoms did not change significantly from the 15th to the 36th month: 12.4% to 10.7% for PTSD (χ^2^ = 0.71, df = 1; p = 0.40), from 13.9% to 13.5% for depression (χ^2^ = 0.04, df = 1; p = 0.84), and from 4.2% to 4.7% for co-occurrence of both symptoms (χ^2^ = 0.12, df = 1; p = 0.73). Figure 2 shows the prevalence rates of PTSD and depression among participants with various traumatic experiences, from which we can see that over 20% of children who had family members or friends seriously injured, had lost family members, or had lost significant others still faced symptoms of PTSD and depression even 36 months after the earthquake.

For both assessments, we found that girls scored significantly higher PTSD scores than boys; children who had witnessed someone being killed or seriously injured, had lost significant others, had lost important belongings, or had felt guilt concerning someone’s death or injury reported higher PTSD scores than those who had not (Table 2). From the 15th to the 36th month after the earthquake, the overall score for both PTSD and depression did not change significantly. But the situation differed, conditional on children’s demographics and traumatic experiences. Children living in Wenchuan experienced a marked decrease of depression score over the time, while children who were from Mao or were ethnic minorities reported increase of the score. Curiously, we found that children who were identified as not having been seriously injured, having lost family members, having lost significant others, or having felt guilt concerning someone’s death or injury reported an increase of PTSD score over time (Table 2).

**Table 2:** Mean score at the 15th month and mean score difference between the 36th and 15th month, for PTSD and depression

	**PTSD**		**Depression**	
	**Month 15**	**Diff (95% CI)**	**Month 15**	**Diff (95% CI)**
Overall	19.0	1.30 (−0.32, 2.91)	9.6	0.19 (−0.72, 1.10)
Demographics				
Residence				
Wenchuan	17.2	1.79 (−0.88, 4.46)	10.3	−2.40 (−3.91, -0.89)**
Mao	20.2	0.87 (−1.14, 2.88)	9.4	1.94 (0.82, 3.05)**
Sex				
Boys	17.7	1.18 (−1.06, 3.42)	9.7	−0.12 (−1.41, 1.18)
Girls	20.3	1.30 (−1.01, 3.61)	9.8	0.48 (−0.80, 1.76)
Ethnicity				
Han	16.6	1.52 (−4.36, 7.39)	10.7	−2.93 (−6.27, 0.42)
Ethnic minorities	20.5	−0.06 (−1.84, 1.72)	9.4	1.08 (0.08, 2.08)*
Earthquake-related experiences				
Having been in serious danger (Eq1)				
Yes	19.9	3.03 (−0.29, 6.36)	9.9	0.70 (−1.11, 2.51)
No	18.1	1.45 (−0.44, 3.33)	9.6	0.10 (−1.00, 1.19)
Having been seriously injured (Eq2)				
Yes	24.7	0.55 (−8.31, 9.41)	12.4	0.85 (−3.86, 5.56)
No	18.4	1.71 (0.09, 3.33)*	9.5	0.33 (−0.59, 1.25)
Having family members or friends seriously injured (Eq3)				
Yes	25.4	1.41 (−3.33, 6.15)	12.3	2.22 (−0.33, 4.77)
No	17.7	1.47 (−0.19, 3.12)	9.2	−0.11 (−1.07, 0.84)
Having witnessed someone being killed or seriously injured (Eq4)				
Yes	23.1	1.42 (−1.50, 4.33)	10.7	1.11 (−0.44, 2.67)
No	16.2	1.62 (−0.14, 3.38)	9.1	−0.31 (−1.41, 0.79)
Having lost family members (Eq5)				
Yes	30.7	−6.93 (−13.6, -0.26)*	13.6	0.02 (−3.72, 3.76)
No	17.5	2.53 (0.93, 4.12)**	9.3	0.39 (−0.52, 1.31)
Having lost significant others (Eq6)				
Yes	28.4	−2.53 (−6.06, 1.00)	11.9	0.01 (−1.97, 2.00)
No	16.0	2.48 (0.83, 4.12)*	9.0	0.23 (−0.78, 1.23)
Having one’s house seriously damaged (Eq7)				
Yes	20.6	1.26 (−0.74, 3.25)	9.8	0.76 (−0.34, 1.87)
No	15.1	2.04 (−0.53, 4.61)	9.5	−1.05 (−2.65, 0.55)
Having lost important belongings (Eq8)				
Yes	22.7	2.32 (−0.54, 5.18)	10.7	0.40 (−1.13, 1.94)
No	16.4	1.39 (−0.44, 3.23)	9.2	0.22 (−0.90, 1.35)
Having felt extremely anxious about one’s own life (Eq9)				
Yes	20.7	1.04 (−0.87, 2.94)	9.9	0.29 (−0.76, 1.34)
No	14.1	1.28 (−1.40, 3.96)	9.2	−0.22 (−2.03, 1.59)
Having felt scared that family members or significant others would die or be seriously injured (Eq10)				
Yes	20.3	1.15 (−0.72, 3.01)	9.8	0.15 (−0.86, 1.17)
No	14.2	2.03 (−0.80, 4.85)	9.6	0.33 (−1.73, 2.39)
Having felt guilt concerning someone’s death or injury (Eq11)				
Yes	24.0	0.21 (−2.76, 3.19)	10.6	0.50 (−1.03, 2.03)
No	16.1	2.04 (0.28, 3.81)*	9.3	0.04 (−1.09, 1.16)

Note: PTSD, posttraumatic stress disorder; CI, confidence interval; PTSD/depression Diff = PTSD/depression score at the 36th month - PTSD/depression score at the 15th month. *P < 0.05; **P < 0.01.

In the study, we found that the proportion of participants who reported earthquake-related utilization of mental health services dropped substantially from 34.6% in the first 15 months to 9.5% in the follow-up period. And Pearson correlations demonstrated the positive role of perceived social support in mitigating the symptoms of PTSD (r = −0.10, p = 0.04) and depression (r = −0.42, p < 0.001). The role of social support in predicting PTSD was no longer significant when entering into the linear regression model with other variables, which is shown in Table 3. The strong correlation between PTSD and depression (r = 0.453; p < 0.001) made them serve as the biggest contributor for each other. A point increase in PTSD or depression was associated with more than 0.40-point increase in the other. As to the other variables, objective experiences, subjective experiences, and earthquake-related loss showed significant contributions to PTSD, while perceived social support showed marked influence on depression.

**Table 3:** Linear regression analysis on the contributions of independent variables to PTSD and depression at the 36th month

	**PTSD (adjusted R**^**2**^ **= 0.389)**			**Depression (adjusted R**^**2**^ **= 0.404)**		
**Variables**^a^	**Beta**	**Partial correlation**	***P***	**Beta**	**Partial correlation**	***P***
PTSD	—	—	—	0.448	0.455	< 0.01
depression	0.462	0.455	< 0.001	—	—	—
Residence	0.033	0.041	0.40	0.062	0.077	0.11
Sex	−0.076	−0.095	0.05	−0.031	−0.039	0.42
Ethnicity	0.006	0.007	0.88	0.053	0.069	0.18
Objective experiences	0.120	0.139	0.004	0.066	0.077	0.11
Subjective experiences	0.182	0.213	< 0.001	−0.033	−0.039	0.43
Earthquake-related loss	0.171	0.192	< 0.001	0.051	0.058	0.24
Perceived social support	0.062	0.070	0.15	−0.364	−0.417	< 0.001

Note. PTSD = posttraumatic stress disorder; Beta = standardized coefficient.

^a^ Residence, sex, and ethnicity were dummy-coded as follows: Wenchuan = 0, Mao = 1; girls = 0, boys = 1; ethnic minorities = 0, Han = 1. Objective experiences (Eq1, Eq2, Eq3 and Eq4), subjective experiences (Eq9, Eq10 and Eq11), and earthquake-related loss (Eq5, Eq6, Eq7 and Eq8) were computed by summing positive responses in each category.

## Discussion

By using a community-based follow-up study, we found that symptoms of PTSD and depression seemed to persist among child and adolescent survivors of the 2008 Sichuan earthquake, and that different traumatic experiences predicted different longitudinal development of PTSD and depression for these children. The study also indicated that the earthquake might have a delayed impact on the psychosocial functioning of children and adolescents who were not directly affected by the disaster.

The persistence of the symptoms could possibly be related to unremitting multiple post-disaster adversities that aggravated the symptoms or impeded their remission [9,18]. Conversely, continued symptoms of PTSD and depression seemed to contribute to secondary stresses and adversities, such as disturbances in school and social functioning, which in turn imposed more difficulties for them to cope with the symptoms. Our study showed that children who had family members or friends seriously injured, had lost family members, or had lost significant others still faced higher prevalence of PTSD and depression than children with other traumatic experiences 36 months after the earthquake. The result may help to screen out children with an elevated risk for persistent psychological problems so that appropriate mental health interventions could be allocated to them.

However, the severity of PTSD differed from previous reports. A study among child survivors after the 1999 Chi-Chi earthquake in Taiwan indicated that victims near the epicenter had moderate levels of PTSD one year after the earthquake [32]; Goenjian et al., using Child PTSD Reaction Index, reported moderate reaction scores in bereaved adolescents six and a half years after the 1988 Spitak earthquake [9], and severe levels of PTSD in adolescents from three public schools six months after hurricane Mitch [33]. In contrast, our study found mild levels of PTSD. The differences may be attributed to different sampling frames. Our study was based on a large community-based sample, while previous studies primarily studied children with specific characteristics or in places with unique features (epicenter, etc.), which might result in a more severe estimation of PTSD symptoms.

A curious point deserves further notice, viz., children who were identified as not having been seriously injured, having lost family members, having lost significant others, or having felt guilt concerning someone’s death or injury reported an increase of PTSD score over the time. A possible speculation is that the earthquake, together with post-earthquake changes in social environment, had a delayed impact on the psychosocial functioning of children and adolescents who were not directly affected by the disaster. For example, while a child who lost his or her parent was likely to receive more support and care from families, relatives, friends and communities after the earthquake, a child without bereavement may get less help. Just as scarce mental health services may be especially allocated to children with more severe traumatic experiences while leaving children without a specific experience unnoticed, children with potential psychological symptoms may be ignored. Thus the finding that earthquake-related mental health utilization fell sharply from 34.6% in the first 15 months to 9.5% in the follow-up period raised great concerns over how to fulfill the unmet needs of psychological recovery for these children.

As the findings from the Japan and Pakistan earthquakes suggested that post-disaster chronic psychological needs were often inadequately managed and could lead to increased rates of complications and indirect morbidity after a disaster [34,35], measures should be taken to ameliorate the persistent symptoms for child and adolescent survivors of the 2008 Sichuan earthquake. Efforts should be made in several ways. First, in order to facilitate the early identification of children with mental health problems, a comprehensive screening program should be established to evaluate the mental health conditions of these children at regular intervals. Schools may serve as an essential role in early identification and could be able to provide psychological interventions for these children. Second, as the children with mental health problems tend not to come for help, community-based mental health support—such as reaching out to them—may be a better option. In addition, all of those efforts should not be segregated from the other interventions, because these children not only have psychological needs but also require physical, economic, spiritual support as well.

### Strengths and limitations

To our knowledge, this is the first community-based study that examines the longitudinal outcomes of mental health among child and adolescent survivors of an earthquake. Although community-based research has gained robust applications in indentifying and solving problems that exist in almost every corner of public health, the combination of a community-based study design and the context of a natural disaster is indeed a bold attempt. A major limitation of the study should also be recognized. All participants in the study experienced the earthquake, and they were not compared with controls from non-affected areas. It was because the Sichuan earthquake was so powerful and extensive that the shock spread over almost half of mainland China, Additionally, it is a challenge to select a significant sample size of children and adolescents who have not experienced the disaster. Even if children were selected as non-exposed, there is significant demographic and cultural variation between exposed and unexposed children. Nevertheless, as the study indicated that children with more earthquake-related experiences predicted more severe PTSD and depression, we are confident to assume that children affected by the earthquake have more severe mental health symptoms than those without the experience.

## Conclusions

Problems that require treatment often do not manifest themselves until much later in life, so primary prevention is essential for young people. Identifying factors that are associated with vulnerability to post-earthquake psychological problems is the beginning for preventing public mental health nightmares in the future. Our results suggested that a substantial proportion of children who had family members or friends seriously injured, had lost family members, or had lost significant others still suffered symptoms of PTSD and depression even three years after the earthquake. We also found that the earthquake might have a delayed impact on the psychosocial functioning of children and adolescents who were not directly affected by the disaster. Despite of the crucial situation, the proportion of participants who reported earthquake-related utilization of mental health services dropped substantially from 34.6% in the first 15 months to 9.5% in the follow-up period, which raised great concerns over how to fulfill the unmet psychological needs of these children.

## Competing interests

The authors declare that they have no competing interests.

## Authors’ contributions

ZJ, LS and WT analysed the data and wrote the manuscript. ZJ, GD, XP and WL collected the data and were involved in critical revisions. WT, LS and YC were involved in conceptualizing and formulating the principal issues. WT and YC supervised the study. ZJ and WT had full access to all of the data in the study and take responsibility for the integrity of the data and the accuracy of the data analysis. WT is guarantor. All authors read and approved the final manuscript.

## Pre-publication history

The pre-publication history for this paper can be accessed here:

http://www.biomedcentral.com/1471-2458/13/104/prepub

## References

[B1] Earthquakes caused the deadliest disasters in the past decadehttp://www.unisdr.org/archive/12470

[B2] Centers for Diseases and Control Rapid health response, assessment, and surveillance after a tsunami--Thailand, 2004–2005 MMWR Morb Mortal Wkly Rep 2005 54 3 61 64 15674183

[B3] Sullivan KM Hossain SM Earthquake mortality in Pakistan Disasters 2010 34 1 176 183 10.1111/j.1467-7717.2009.01121.x 19682002

[B4] Jia Z Tian W He X Liu W Jin C Ding H Mental health and quality of life survey among child survivors of the 2008 Sichuan earthquake Qual Life Res 2010 19 9 1381 1391 10.1007/s11136-010-9703-8 20623339

[B5] Damage Situation and Police Countermeasures associated with 2011 Tohoku districthttp://www.npa.go.jp/archive/keibi/biki/higaijokyo_e.pdf

[B6] Eksi A Braun KL Over-time changes in PTSD and depression among children surviving the 1999 Istanbul earthquake Eur Child Adolesc Psychiatry 2009 18 6 384 391 10.1007/s00787-009-0745-9 19221855

[B7] Goenjian AK Roussos A Steinberg AM Sotiropoulou C Walling D Kakaki M Karagianni S Longitudinal study of PTSD, depression, and quality of life among adolescents after the Parnitha earthquake J Affect Disord 2011 133 509 515 10.1016/j.jad.2011.04.053 21641650

[B8] Goenjian AK Walling D Steinberg AM Karayan I Najarian LM Pynoos R A prospective study of posttraumatic stress and depressive reactions among treated and untreated adolescents 5 years after a catastrophic disaster Am J Psychiatry 2005 162 12 2302 2308 10.1176/appi.ajp.162.12.2302 16330594

[B9] Goenjian AK Walling D Steinberg AM Roussos A Goenjian HA Pynoos RS Depression and PTSD symptoms among bereaved adolescents 6(1/2) years after the 1988 Spitak earthquake J Affect Disord 2009 112 1–3 81 84 18547646 10.1016/j.jad.2008.04.006

[B10] Roussos A Goenjian AK Steinberg AM Sotiropoulou C Kakaki M Kabakos C Karagianni S Manouras V Posttraumatic stress and depressive reactions among children and adolescents after the 1999 earthquake in Ano Liosia, Greece Am J Psychiatry 2005 162 3 530 537 10.1176/appi.ajp.162.3.530 15741470

[B11] Hsu CC Chong MY Yang P Yen CF Posttraumatic stress disorder among adolescent earthquake victims in Taiwan J Am Acad Child Adolesc Psychiatry 2002 41 7 875 881 10.1097/00004583-200207000-00022 12108814

[B12] Poli P Sbrana B Marcheschi M Masi G Self-reported depressive symptoms in a school sample of Italian children and adolescents Child Psychiatry Hum Dev 2003 33 3 209 226 10.1023/A:1021404613832 12564623

[B13] Kolaitis G Kotsopoulos J Tsiantis J Haritaki S Rigizou F Zacharaki L Riga E Augoustatou A Bimbou A Kanari N Posttraumatic stress reactions among children following the Athens earthquake of September 1999 Eur Child Adolesc Psychiatry 2003 12(6 273 280 14689259 10.1007/s00787-003-0339-x

[B14] Lee I Ha YS Kim YA Kwon YH PTSD symptoms in elementary school children after Typhoon Rusa Taehan Kanho Hakhoe Chi 2004 34 4 636 645 15502429 10.4040/jkan.2004.34.4.636

[B15] Awasthi S Agnihotri K Singh U Thakur S Chandra H Determinants of health related quality of life in school-going adolescents in Northern India Indian J Pediatr 2011 78 5 555 561 10.1007/s12098-010-0305-9 21267797

[B16] Yang S An analysis of the status of out-of-school children in China China Popul Today 1996 13 4 11 13 12320580

[B17] Liu M Wang L Shi Z Zhang Z Zhang K Shen J Mental health problems among children one-year after Sichuan earthquake in China: a follow-up study PLoS One 2011 6 2 e14706 10.1371/journal.pone.0014706 21373188 PMC3044135

[B18] Thienkrua W Cardozo BL Chakkraband ML Guadamuz TE Pengjuntr W Tantipiwatanaskul P Sakornsatian S Ekassawin S Panyayong B Varangrat A Symptoms of posttraumatic stress disorder and depression among children in tsunami-affected areas in southern Thailand JAMA 2006 296 5 549 559 10.1001/jama.296.5.549 16882961

[B19] Stacciarini JM Shattell MM Coady M Wiens B Review: community-based participatory research approach to address mental health in minority populations Community Ment Health J 2011 47 5 489 497 10.1007/s10597-010-9319-z 20464489

[B20] Kish L A procedure for objective respondent selection within the household J Am Stat Assoc 1949 44 380 387 10.1080/01621459.1949.10483314

[B21] Pynoos RS Frederick C Nader K Arroyo W Steinberg A Eth S Nunez F Fairbanks L Life threat and posttraumatic stress in school-age children Arch Gen Psychiatry 1987 44 12 1057 1063 10.1001/archpsyc.1987.01800240031005 3689093

[B22] Steinberg AM Brymer MJ Decker KB Pynoos RS The University of California at Los Angeles Post-traumatic Stress Disorder Reaction Index Curr Psychiatry Rep 2004 6 2 96 100 10.1007/s11920-004-0048-2 15038911

[B23] Hong X Li J Xu F Tse LA Liang Y Wang Z Yu IT Griffiths S Physical activity inversely associated with the presence of depression among urban adolescents in regional China BMC Publ Health 2009 9 148 10.1186/1471-2458-9-148 PMC2693135 19457241

[B24] Kovacs M Rating scales to assess depression in school-aged children Acta Paedopsychiatr 1981 46 5–6 305 315 7025571

[B25] Chang HJ Yang CY Lin CR Ku YL Lee MB Determinants of suicidal ideation in Taiwanese urban adolescents J Formos Med Assoc 2008 107 2 156 164 10.1016/S0929-6646(08)60129-1 18285248

[B26] Chao CC Chen SH Wang CY Wu YC Yeh CH Psychosocial adjustment among pediatric cancer patients and their parents Psychiatry Clin Neurosci 2003 57 1 75 81 10.1046/j.1440-1819.2003.01082.x 12519458

[B27] Yu D Li X Preliminary use of the Children’s Depression Inventory in China Zhong Guo Xin Li Wei Sheng Za Zhi 2000 14 4 225 227

[B28] Bruwer B Emsley R Kidd M Lochner C Seedat S Psychometric properties of the Multidimensional Scale of Perceived Social Support in youth Compr Psychiatry 2008 49 2 195 201 10.1016/j.comppsych.2007.09.002 18243894

[B29] Zimet GD Powell SS Farley GK Werkman S Berkoff KA Psychometric characteristics of the Multidimensional Scale of Perceived Social Support J Pers Assess 1990 55 3–4 610 617 2280326 10.1080/00223891.1990.9674095

[B30] Hong Y Li X Fang X Zhao G Lin X Zhang J Zhao J Zhang L Perceived social support and psychosocial distress among children affected by AIDS in china Community Ment Health J 2010 46 1 33 43 10.1007/s10597-009-9201-z 19533349 PMC8185877

[B31] McWilliams LA Cox BJ Enns MW Clara IP Personality correlates of outpatient mental health service utilization: findings from the U.S. national comorbidity survey Soc Psychiatry Psychiatr Epidemiol 2006 41 5 357 363 10.1007/s00127-006-0040-8 16565922

[B32] Chen S Lin Y Tseng H Wu Y Posttraumatic stress reactions in children and adolescents one year after the 1999 Taiwan Chi-Chi earthquake J Chin Inst Eng 2002 25 5 597 608 10.1080/02533839.2002.9670734

[B33] Goenjian AK Molina L Steinberg AM Fairbanks LA Alvarez ML Goenjian HA Pynoos RS Posttraumatic stress and depressive reactions among Nicaraguan adolescents after hurricane Mitch Am J Psychiatry 2001 158 5 788 794 10.1176/appi.ajp.158.5.788 11329403

[B34] Matsuoka T Yoshioka T Oda J Tanaka H Kuwagata Y Sugimoto H Sugimoto T The impact of a catastrophic earthquake on morbidity rates for various illnesses Public Health 2000 114 4 249 253 10962585 10.1038/sj.ph.1900660

[B35] Miller AC Arquilla B Chronic diseases and natural hazards: impact of disasters on diabetic, renal, and cardiac patients Prehosp Disaster Med 2008 23 2 185 194 18557300 10.1017/s1049023x00005835

